# *Hox11* genes regulate postnatal longitudinal bone growth and growth plate proliferation

**DOI:** 10.1242/bio.012500

**Published:** 2015-10-23

**Authors:** Kyriel M. Pineault, Ilea T. Swinehart, Kayla N. Garthus, Edward Ho, Qing Yao, Ernestina Schipani, Kenneth M. Kozloff, Deneen M. Wellik

**Affiliations:** 1 Program in Cell and Developmental Biology, University of Michigan, Ann Arbor, MI 48109-2200, USA; 2 Department of Internal Medicine, Division of Molecular Medicine and Genetics, University of Michigan, Ann Arbor, MI 48109-2200, USA; 3 Department of Orthopaedic Surgery, University of Michigan, Ann Arbor, MI 48109, USA

**Keywords:** Hox genes, Growth plate, Chondrocyte, Postnatal skeletal development

## Abstract

Hox genes are critical regulators of skeletal development and *Hox9*-*13* paralogs, specifically, are necessary for appendicular development along the proximal to distal axis. Loss of function of both *Hoxa11* and *Hoxd11* results in severe malformation of the forelimb zeugopod. In the radius and ulna of these mutants, chondrocyte development is perturbed, growth plates are not established, and skeletal growth and maturation fails. In compound mutants in which one of the four *Hox11* alleles remains wild-type, establishment of a growth plate is preserved and embryos develop normally through newborn stages, however, skeletal phenotypes become evident postnatally. During postnatal development, the radial and ulnar growth rate slows compared to wild-type controls and terminal bone length is reduced. Growth plate height is decreased in mutants and premature growth plate senescence occurs along with abnormally high levels of chondrocyte proliferation in the reserve and proliferative zones. Compound mutants additionally develop an abnormal curvature of the radius, which causes significant distortion of the carpal elements. The progressive bowing of the radius appears to result from physical constraint caused by the disproportionately slower growth of the ulna than the radius. Collectively, these data are consistent with premature depletion of forelimb zeugopod progenitor cells in the growth plate of *Hox11* compound mutants, and demonstrate a continued function for *Hox* genes in postnatal bone growth and patterning.

## Abstract (precis)

**Summary:** Hox genes are required for postnatal skeletal growth. *Hox11* genes function in the zeugopod skeletal elements and regulate longitudinal growth through controlling chondrocyte maturation within the growth plate.

## INTRODUCTION

The proximodistal elongation of long bones occurs through the process of endochondral ossification within the growth plate. The growth plate is organized into distinct cellular zones: the reserve or resting zone (RZ), the proliferative zone (PZ), and the prehypertrophic/hypertrophic zone (HZ). Chondrocytes at the epiphyseal end of the growth plate make up the reserve zone. RZ chondrocytes are slow dividing, stem/progenitor cells which give rise to flattened columns of PZ chondrocytes (Abad et al., 2002). Chondrocytes in the PZ proliferate rapidly and undergo morphological changes to become oriented into columns of cells arranged parallel to the long axis of the bone (Hunziker, 1994). As cells in the columns reach the metaphyseal side of the growth plate and enter the HZ, it has been reported that chondrocytes exit the cell cycle, undergo hypertrophy and initiate apoptosis (Beier, 2005). Concomitantly, hypertrophic chondrocytes secrete significant amounts of extracellular matrix which becomes mineralized and provides a template for new bone formation (Mackie et al., 2011). The cartilage matrix of the growth plate is subsequently remodeled and replaced by bone through the function of osteoclasts and osteoblasts.

Longitudinal bone growth is regulated both locally and systemically by a host of hormones and growth factors that function to coordinate proliferation, matrix synthesis and differentiation of chondrocytes (Kronenberg, 2003; Wit and Camacho-Hubner, 2011). While the differentiation program of growth plate chondrocytes is similar in all long bones, elongation occurs at different rates for each element to achieve correct proportionate lengths for the adult animal (Wilsman et al., 1996b). These rates of growth can vary dramatically. Indeed, growth plates at opposite ends of the same long bone can differ by a factor of two- to three-fold (Wilsman et al., 1996b). These differences in growth rate contribute to final bone lengths and morphology, however the processes that govern these differential rates of growth are poorly understood.

*Hox* genes are a family of highly conserved, homeodomain-containing transcription factors crucial for axial and appendicular patterning. In the limb skeleton, *Hox9*-*13* genes function to pattern the proximodistal axis and loss-of-function mutations of paralogous genes result in dramatic, region-specific perturbations of skeletal development. Loss of *Hox9* and/or *Hox10* genes results in mispatterning primarily of the stylopod element (humerus or femur) (Fromental-Ramain et al., 1996b; Wellik and Capecchi, 2003). Loss of *Hox11* function leads to severely truncated zeugopod skeletal elements (radius/ulna or tibia/fibula) (Davis et al., 1995; Wellik and Capecchi, 2003; Boulet and Capecchi, 2004). In *Hoxa13/Hoxd13* mutants, the autopod skeletal elements (hand or foot bones) are affected (Dollé et al., 1993; Fromental-Ramain et al., 1996a; Knosp et al., 2004). The genetic function of *Hox* factors during development is well documented, but continuing roles for these genes postnatally is less explored.

A high degree of functional redundancy exists between members of a *Hox* paralogous group. For example, single allele mutants of *Hoxa11* or *Hoxd11* exhibit minor developmental defects, which include fusion of various carpal bones, minor malformations of the distal epiphyseal end and a slight thickening of the radius and ulna (Small and Potter, 1993; Davis and Capecchi, 1994; Favier et al., 1995). These phenotypes are in stark contrast to the dramatic mispatterning observed when all *Hox11* paralogous genes are lost (*Hoxa11^−/−^;Hoxd11^−/−^* in the forelimb or *Hoxa11^−/−^;Hoxc11^−/−^;Hoxd11^−/−^* in the hindlimb). In these mutants, the cartilage anlage condense normally, however, the subsequent maturation of the chondrocytes fails, an organized growth plate is not established and no skeletal pattern is elaborated (Boulet and Capecchi, 2004). Interestingly, maintenance of a single functional allele of *Hoxa11* or *Hoxd11* is sufficient to allow for normal embryonic skeletal development in the forelimb (Davis et al., 1995; Boulet and Capecchi, 2004; Swinehart et al., 2013). In these *Hox11* compound mutant embryos, no differences in proliferation, apoptosis, or overall skeletal growth was observed (Boulet and Capecchi, 2004). However, by adult stages, *Hox11* compound mutant animals exhibit a significant reduction in zeugopod skeletal length (Davis et al., 1995). The purpose of this study was to define the morphological and cellular processes that contribute to the postnatal growth defects in *Hox11* compound mutants during postnatal development.

We show that *Hoxa11* and *Hoxd11* continue to be expressed in the forelimb zeugopod throughout postnatal stages. Consistent with previous reports, the skeletal morphology of *Hox11* compound mutants is indistinguishable from controls at birth. However, during postnatal growth, the compound mutant radius and ulna grow at slower rates than controls at all time points examined and growth arrests at earlier stages. Despite total bone lengths being comparable to controls at birth, growth plate length is shortened in compound mutants. Chondrocytes are prematurely depleted in the mutant growth plate during postnatal growth correlated with increased levels of proliferation in the RZ and PZ. Additionally, the longitudinal growth of the compound mutant ulna is more severely affected than the radius resulting in a bone that is disproportionately shorter. The altered proportions of the radius and ulna contribute to an anterior bowing of the compound mutant radius in adult animals. Together, these results demonstrate a continuing role for *Hox11* genes in postnatal bone growth.

## RESULTS

### *Hox11* genes remain expressed through postnatal and adult stages

**Fig. 1.**
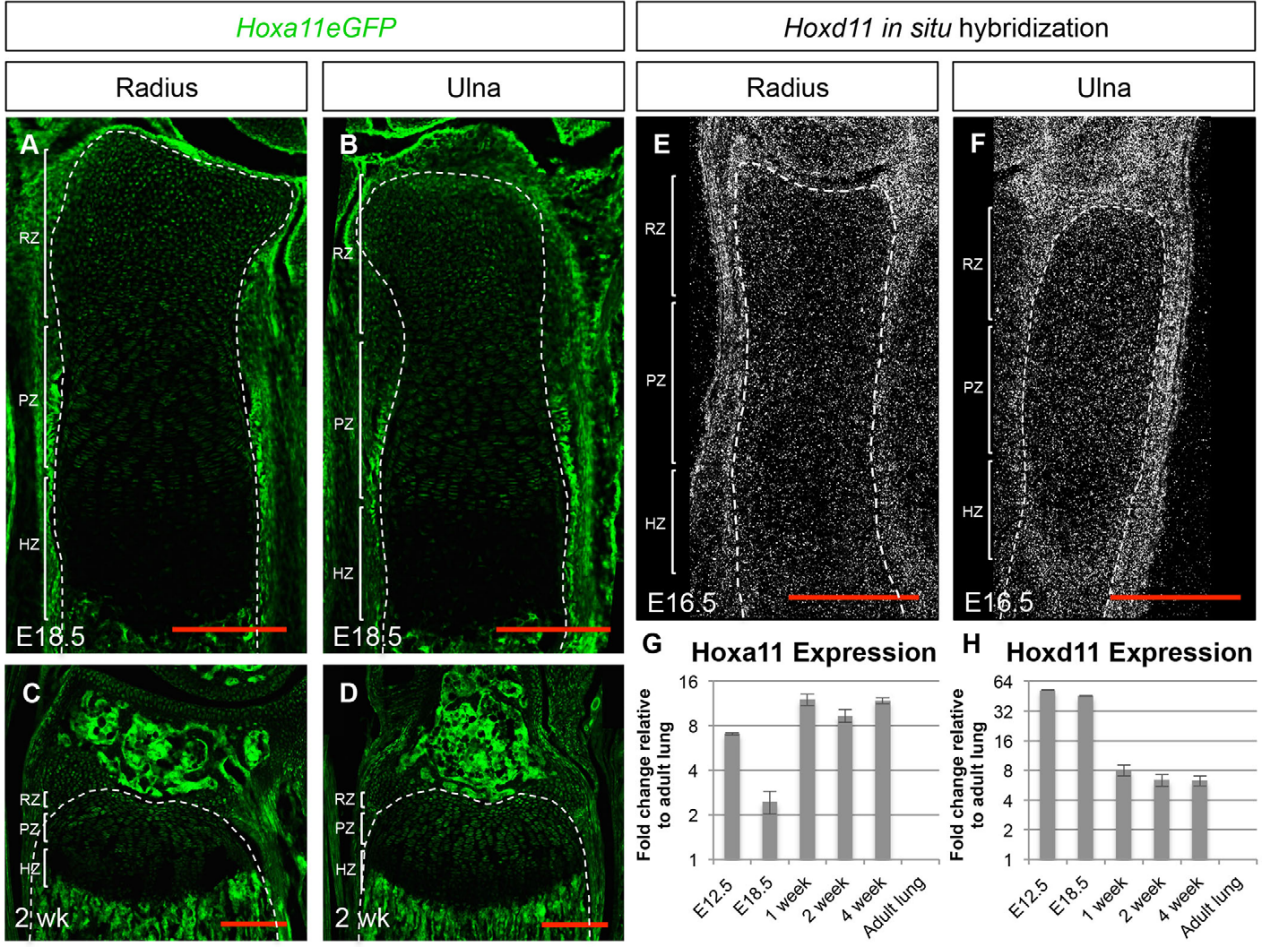
**Expression of *Hoxa11* and *Hoxd11* in post-natal development.** (A-D) Expression of *Hoxa11eGFP* in E18.5 radius (A) and ulna (B) and in 2-week old radius (C) and ulna (D). (E,F) *In situ* hybridization of *Hoxd11* in E16.5 radius (E) and ulna (F). Dashed white line outlines the distal growth plate and brackets demarcate the zones of the growth plate. (G) qPCR analysis of *Hoxa11* expression at E12.5, 18.5 and 1, 2, and 4 weeks of age. (H) qPCR analysis of *Hoxd11* expression at E12.5, E18.5 and at 1, 2, and 4 weeks of age. Expression values in G and H are relative to *Hoxa11* or *Hoxd11* expression in adult lung, which is set to ‘1’. Error bars depict standard deviation. RZ, reserve zone; PZ, proliferative zone; HZ, hypertrophic zone.

We previously published detailed developmental expression analysis of *Hoxa11* in the embryo utilizing a *Hoxa11eGFP* knock-in allele (Nelson et al., 2008; Swinehart et al., 2013). Embryonically, *Hoxa11eGFP* is expressed in the connective tissue elements of the forelimb including the perichondrium/periosteum, tendon and muscle connective tissue with the strongest expression surrounding the distal end of the radius and ulna (Swinehart et al., 2013). Utilizing this *Hoxa11eGFP* allele, we show that *Hoxa11eGFP* continues to be expressed at birth and through postnatal stages. *Hoxa11eGFP* is expressed strongly in the perichondrium and along the trabecular bone surface, with lower expression observed within the most distal RZ chondrocytes (Fig. 1A-D). Expression of *Hoxd11* is observed in the connective tissue surrounding the distal radius and ulna in a pattern similar to *Hoxa11eGFP* expression (Fig. 1E-F and data not shown). qPCR analysis for both *Hoxa11* and *Hoxd11* on whole bones (radius and ulna) at E12.5, E18.5 and 1, 2, and 4 weeks of age demonstrates continued expression of both genes through adult stages. Interestingly, *Hoxd11* shows higher relative expression at embryonic stages, but both *Hoxa11* and *Hoxd11* remain expressed through adult stages.

### Abnormal postnatal skeletal growth in *Hox11* compound mutants

**Fig. 2.**
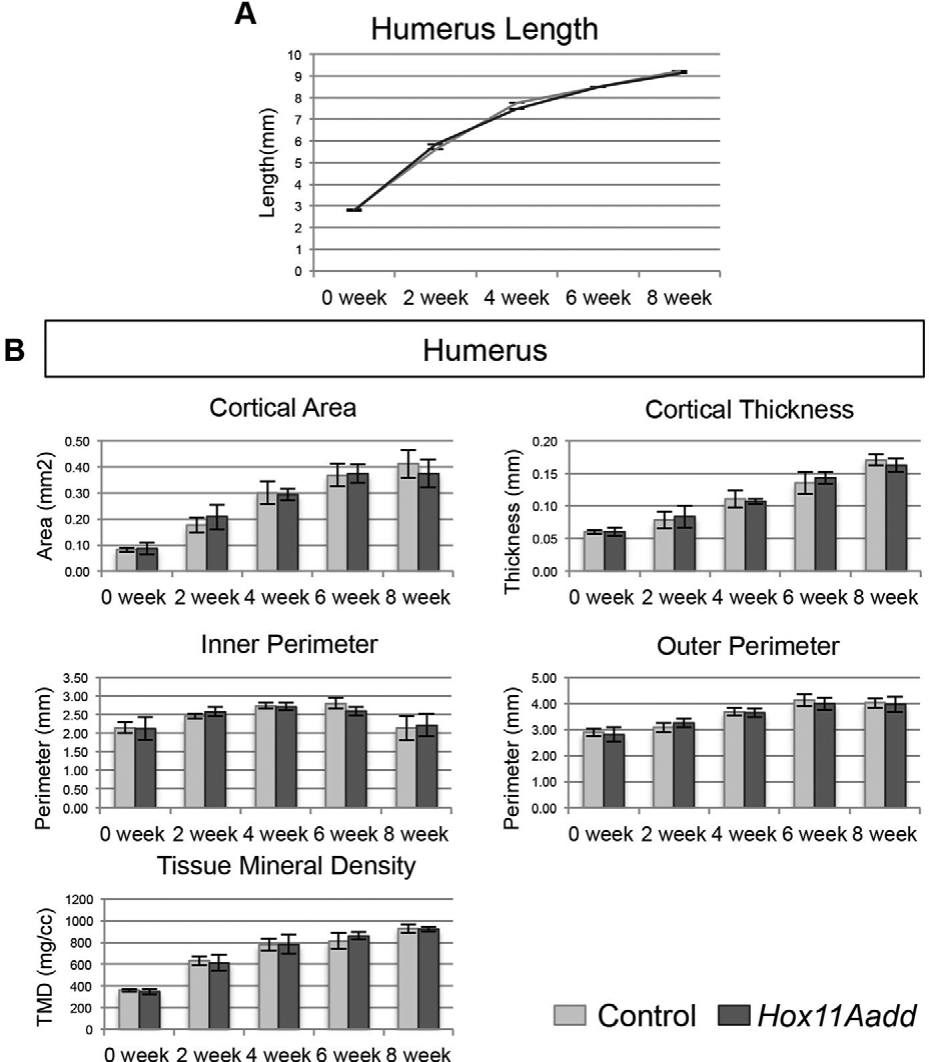
**Development of the humerus is unaffected in *Hox11* compound mutants.** (A) Mineralized length of the humerus in control (light grey) and *Hox11* compound mutant (dark grey) at 0, 2, 4, 6, and 8 weeks of age was measured along the central curvature of the element. (B) µCT analysis of bone morphology and quality of the humerus of control (light grey) and *Hox11* compound mutant (dark grey). Cortical area and thickness, inner and outer perimeter, and tissue mineral density was measured at 0, 2, 4, 6, and 8 weeks of age. Error bars depict standard deviation.

**Fig. 3.**
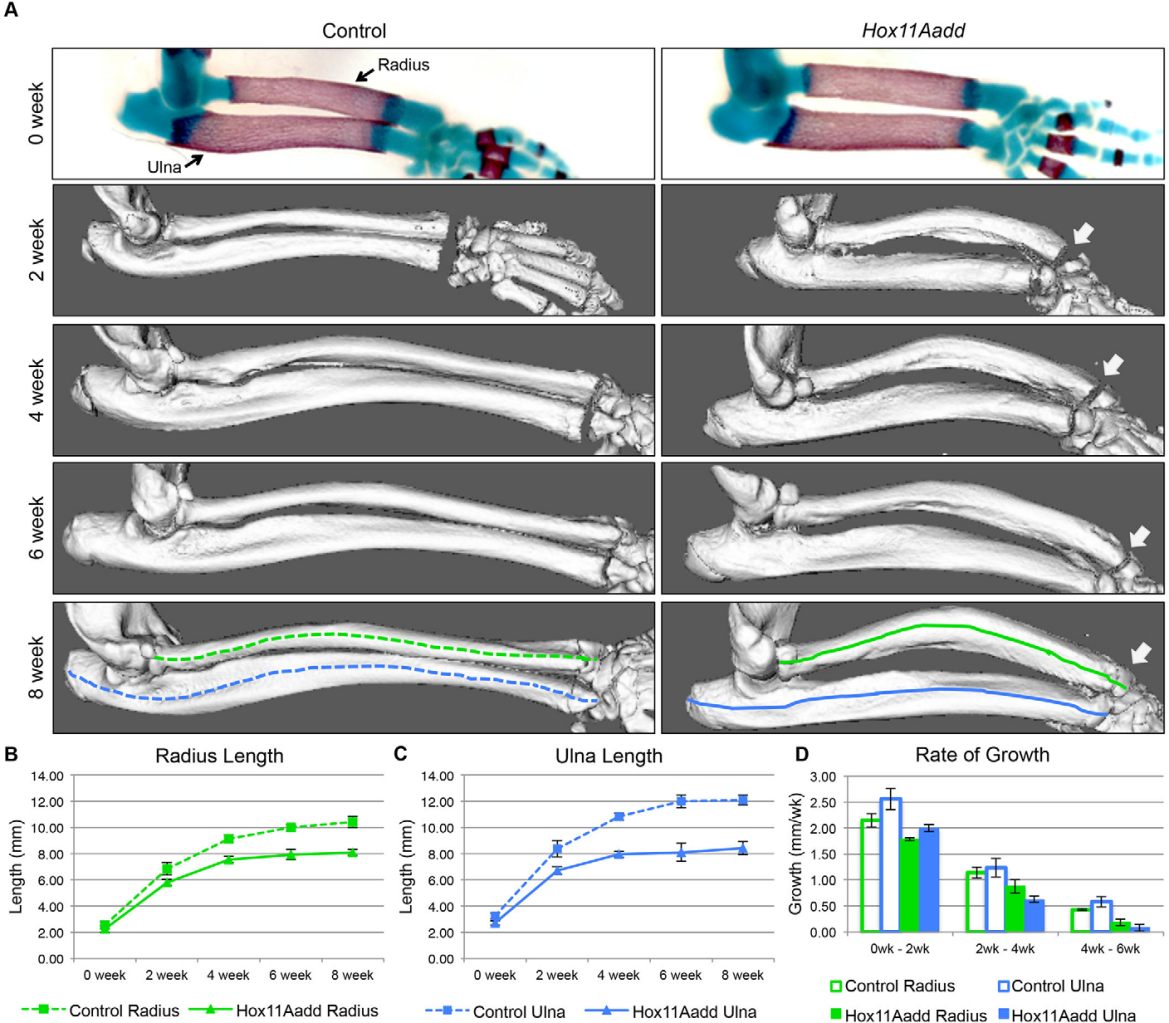
**Postnatal development of wild-type control and *Hox11* compound mutant forelimb zeugopod.** (A) Skeletal preparation of newborn (0 week) forelimb and 3D rendering of µCT scans of 2, 4, 6 and 8 week forelimb from control and *Hox11* compound mutant. Dashed and solid lines indicate central curvature of the element. White arrows highlight distortion of the carpal bones by the radius in the compound mutant. (B,C) Mineralized length of the radius (B) and ulna (C) at 0, 2, 4, 6 and 8 weeks of age was measured along the central curvature of the element. (D) The rate of growth of the radius and ulna of controls and compound mutants was calculated between 0-2 week, 2-4 week, and 4-6 week of age. Error bars depict standard deviation.

To assess the development of the compound mutant skeletal phenotype during postnatal stages, we performed micro-computed tomography (µCT) analysis at 0, 2, 4, 6 and 8 weeks of age on animals with the genotype: *Hoxa11^+/−^;Hoxd11^−/−^*. The humerus was measured as an internal control for animal size as the length, bone quality, and bone morphology of the humerus and was indistinguishable between *Hox11* compound mutants and controls (Fig. 2). Skeletal preparations and isosurface renderings of µCT scans allow visualization of the morphology of the radius and ulna of control and *Hox11* compound mutants throughout postnatal growth (Fig. 3A). While no statistical difference in mineralized bone length is measurable at birth, the length of the ulna is trending towards being shorter in mutants compared to controls and slight morphological differences in the radius and ulna can already be observed (Fig. 3A-C). Abnormal curvature of the radius in compound mutants is readily apparent at two weeks of age and becomes progressively more severe through postnatal development. In addition, the distal radioulnar joint is dysmorphic and the radius of compound mutants progressively extends ectopically into the carpal bones (Fig. 3A, arrows). The overgrowth of the radius results in a forward subluxation of the hand and outwards rotation of the autopod.

To begin to dissect the onset of the postnatal phenotypes, the mineralized lengths of the radius and ulna were measured along the central curvature of the bone at 0, 2, 4, 6, and 8 weeks of age (Fig. 3A, solid and dashed lines). No significant differences in mineralized length of the radius or ulna was observed at newborn stages between controls and compound mutants, consistent with previous reports (Fig. 3B,C) (Boulet and Capecchi, 2004). Differences in bone length are observed early in postnatal development and by 2 weeks of age, both the radius and ulna of the compound mutant are shorter compared to controls: the radius is 15% reduced and ulna 20% reduced. By 4 weeks of age, the differences in length between the compound mutant and controls are exacerbated further (radius 17%, ulna 27% reduced). Longitudinal bone growth has slowed dramatically or stopped in all bones in both controls and mutants by 8 weeks of age, and at this stage the *Hox11* mutant radius is 22% shorter and the ulna is 30% shorter than their analogous control counterparts (Fig. 3B,C).

In both controls and mutants, the rate of bone growth decreases with age. Consistent with the reduction in total bone length, the growth rate of both the mutant radius and ulna is reduced compared to controls at all time points examined (Fig. 3D, solid bars versus open bars). Interestingly, the rate of growth of the mutant ulna slows faster than the mutant radius, and growth of both elements has essentially stopped by 6 weeks of age in mutants (Fig. 3D, solid blue bars versus solid green bar). While the compound mutant radius grows slower than controls throughout development, the decrease in growth rate over time is comparable to controls (Fig. 3D, solid green bar versus open green bar).

### Bone mineral quality is not affected in *Hox11* compound mutants

**Fig. 4.**
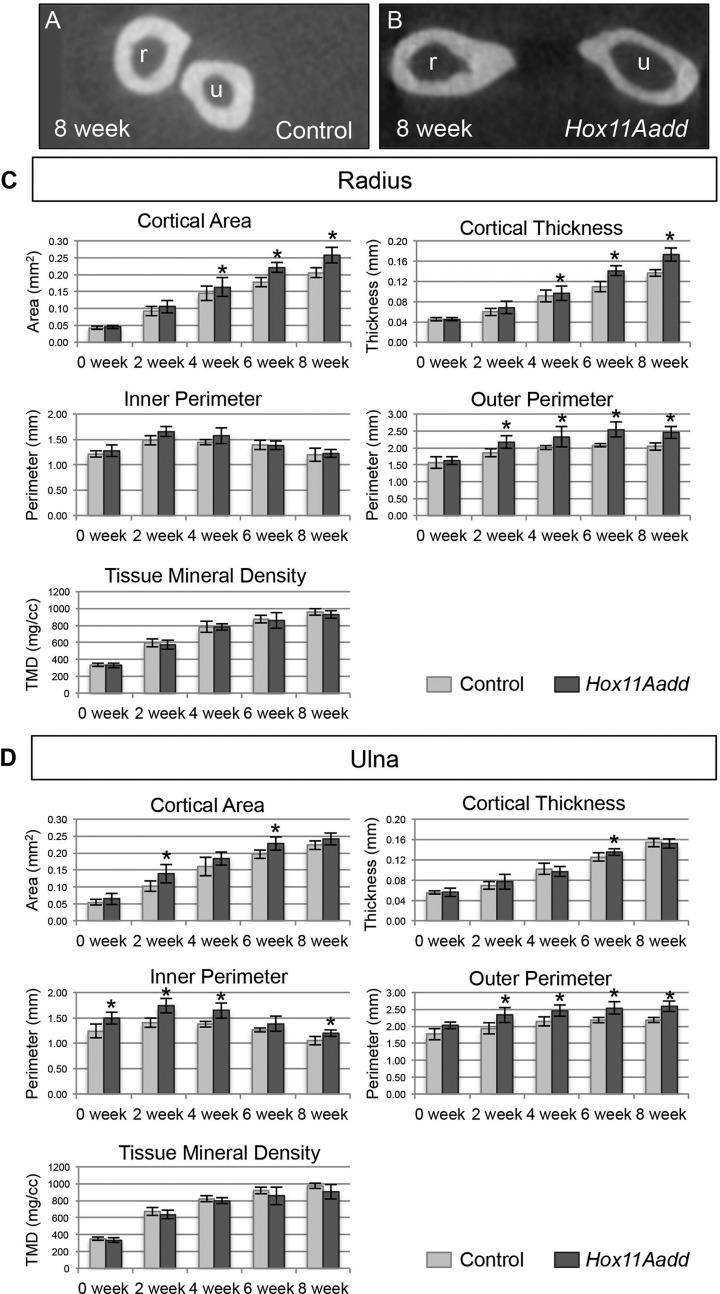
**µCT analysis of bone morphology and quality.** (A,B) Cross-sectional view of µCT scans at 8 weeks of age for control (A) and *Hox11* compound mutant (B) forelimb zeugopod. (C,D) Cortical area and thickness, inner and outer perimeter, and tissue mineral density of the radius (C) and ulna (D) of control (light grey) and compound mutant (dark grey) animals were measured at 0, 2, 4, 6 and 8 weeks of age. *P<0.05; error bars depict standard deviation.

Bone quality of both *Hox11* compound mutants and controls was analyzed by µCT analyses at 0, 2, 4, 6 and 8 weeks of age. No differences in any of the parameters measured, including cortical area and thickness, inner and outer perimeter, and tissue mineral density were observed in the humerus of *Hox11* mutants (Fig. 2B). In contrast, cross-sectional views of the radius and ulna through the mid-diaphysis of control and *Hox11* mutants at 8 weeks of age highlight morphological differences in these animals. Note the bowing phenotype leads to a larger distance between the radius and ulna in compound mutants compared to controls (Fig. 4A,B). Compound mutant bones show measured increases in cortical area, cortical thickness, and bone perimeter compared to controls (Fig. 4C,D). No differences in tissue mineral density are observed between control and *Hox11* compound mutants demonstrating that, despite differences in bone thickness and size, by this measure bone quality appears to be comparable (Fig. 4C,D).

### Appositional bone growth is not coordinated in *Hox11* compound mutant radius and ulna

To investigate appositional growth patterns, we utilized dynamic histomorphometry in the radius and ulna of control and compound mutants. New mineral deposition was analyzed by injection of dye at 2 weeks (xylenol orange label), 3 weeks (calcein label), and 4 weeks (alizarin complexone label) of age. Animals were assessed two days after the last injection. Growth patterns of control and compound mutants were analyzed at the mid-diaphysis where peak curvature is observed in mutant radii. Both xylenol orange (2 weeks) and alizarin complexone (4 weeks) are detected in the red channel, and new bone formed at these two time points can be distinguished by the presence of a green calcein label between the two labels. Rapid growth rates and high bone turnover make quantification of appositional growth rates unfeasible at these time points, however, qualitative differences in overall growth patterns between *Hox11* compound mutants and controls can be assessed.

**Fig. 5.**
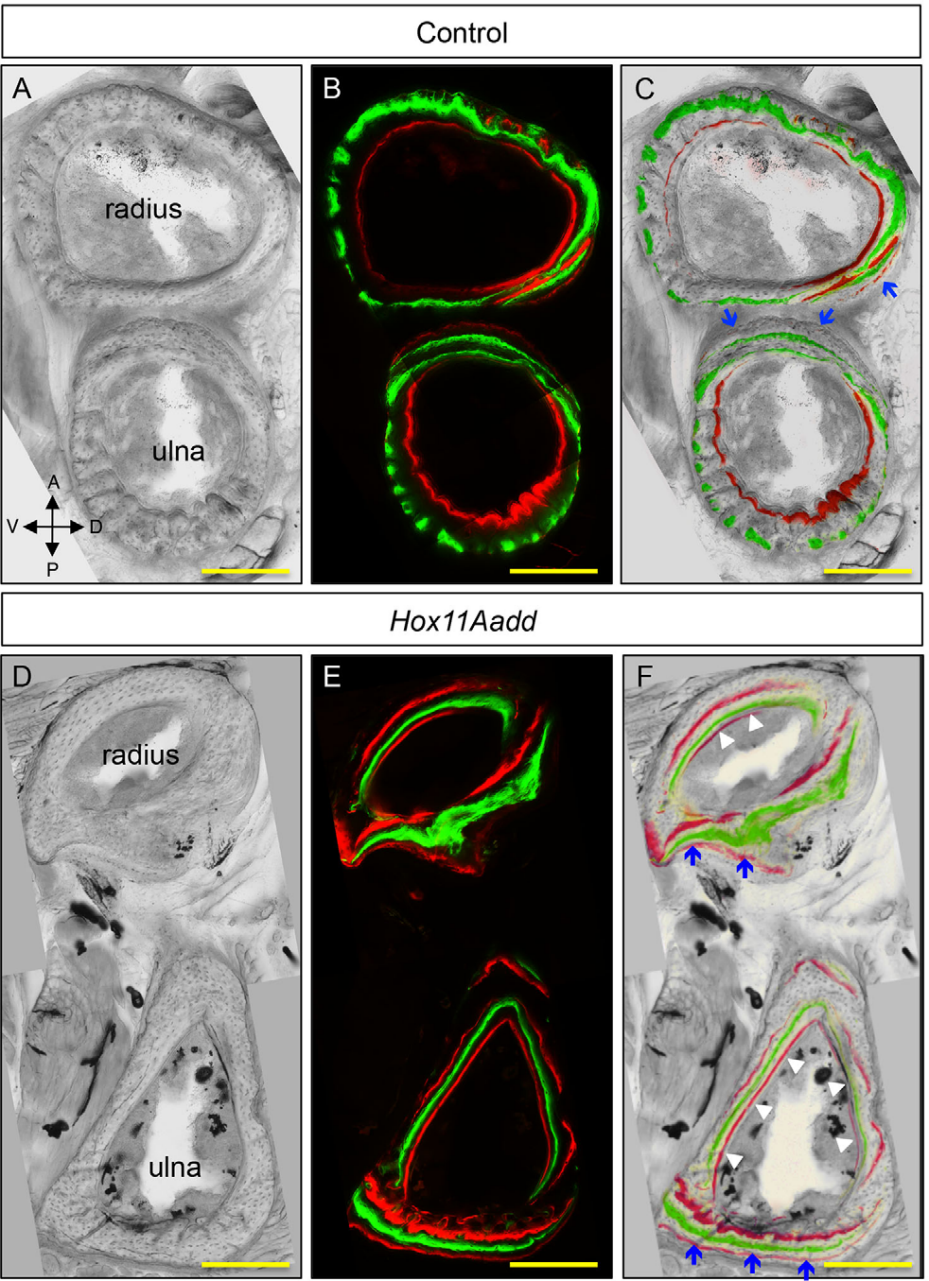
**Appositional bone growth examined by dynamic histomorphometry.** Wild-type control (A-C) and *Hox11* compound mutant (D-F) animals were injected with xylenol orange (red), calcein (green), and alizarin complexone (red) at 2 weeks, 3 weeks and 4 weeks of age respectively. Representative bright field (A,D) and fluorescent (B,E) cross-sectional images of the mid-diaphysis were overlaid (C,F) to examine appositional growth patterns. Areas of appositional growth of the periosteal (blue arrows) and endosteal (white arrowheads) bone surface are highlighted. Scale bar 100 µm.

Within the cortical bone of wild-type controls, there are relatively large distances between labels indicating significant amounts of growth between time points (Fig. 5A-C). Additionally, the distance between labels is not uniform, highlighting the dynamic nature of postnatal skeletal growth. The 3-week label (green) is present as a mostly uninterrupted layer throughout both zeugopod bones, the 4-week label (surface red layer) is observed uniformly on the endosteum of both bones in the control, while the 2-week label (cortical red label) has been largely remodeled away by the time point of analyses (Fig. 5A-C). Appositional growth in control animals is observed only on the posterior-distal periosteal surface of the radius and the anterior periosteal surface of the ulna corresponding to ridges that are being patterned on the bone surface (Fig. 5A-C, blue arrows).

In contrast to control animals, appositional growth in *Hox11* compound mutants is consistently unidirectional in both the radius and ulna. Posterior growth is observed endosteally (Fig. 5D-F, white arrowheads) and periosteally (Fig. 5D-F, blue arrows) in both the radius and ulna. This strong unidirectional growth pattern is established by 2 weeks of age and continues through 4 weeks as demonstrated by incorporation and retention of all three labels (Fig. 5D-F). Minimal incorporation of label on the anterior surface of the compound mutant radius and ulna indicates a lack of new bone formation or high levels of remodeling on this surface. Taken together, the data demonstrate that *Hox11* mutants enact active bone growth in opposition to the direction of anterior radial bowing, consistent with correctional growth.

### *Hox11* compound mutant growth plates are morphologically abnormal and chondrocytes are prematurely depleted

**Fig. 6.**
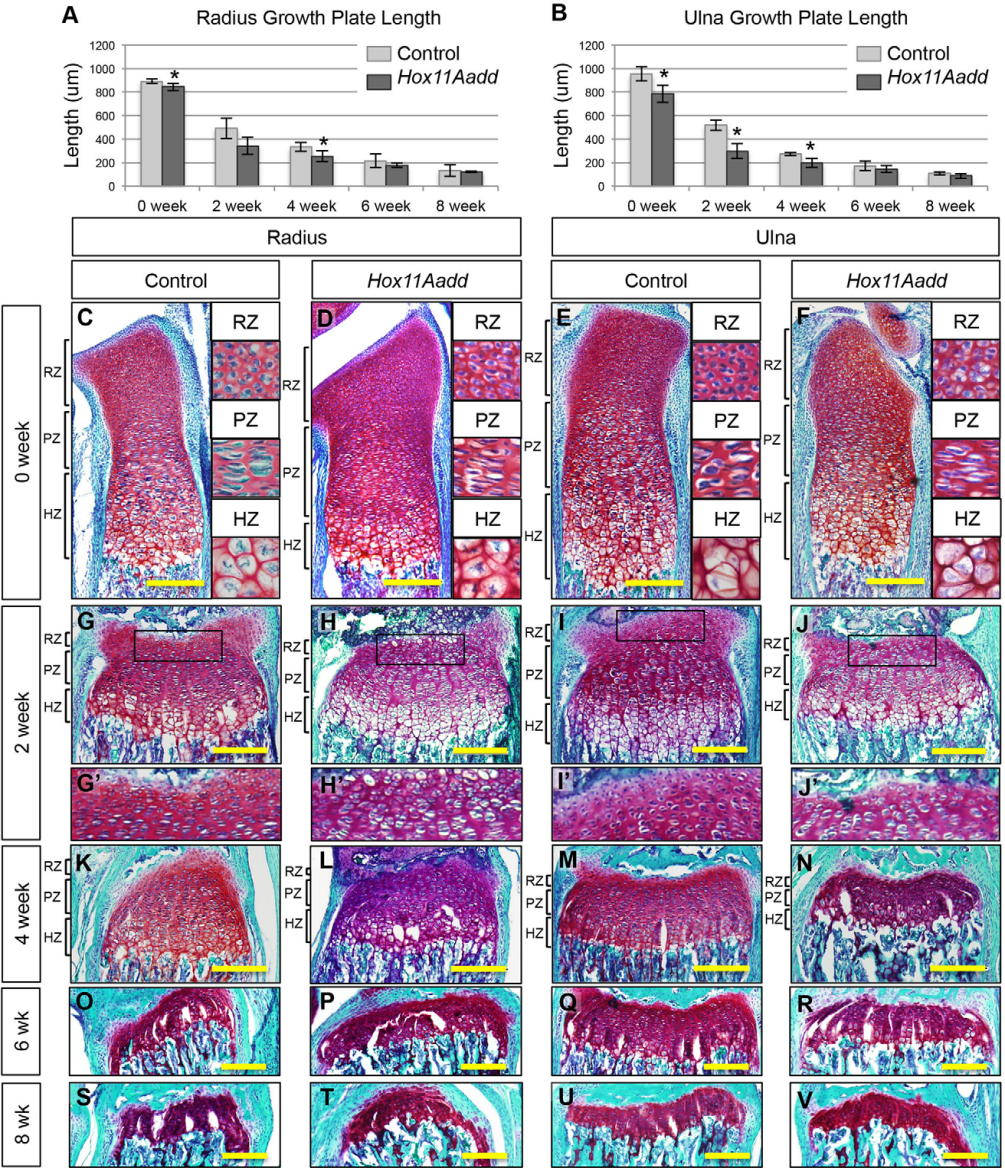
**The distal growth plate of *Hox11* compound mutant forelimb zeugopod bones undergoes premature senescence.** (A-B) Total growth plate length of the radius (A) and ulna (B) from control (light grey) and compound mutant (dark grey) animals was measured from Safranin-O/Fast Green stained sections. Representative images of Safranin-O/Fast Green/Hematoxylin stained distal forelimb growth plates at 0 week (C-F), 2 week (G-J′), 4 week (K-N), 6 week (O-R) and 8 week (S-V) old controls and compound mutants. Black brackets demarcate zones of the growth plate at 0, 2, and 4 weeks (C-J). RZ, reserve zone; PZ, proliferative zone; HZ, hypertrophic zone. *P<0.05; error bars depict standard deviation. Scale bar 200 µm.

Growth plate morphology was analyzed at 0, 2, 4, 6, and 8 weeks of age in controls and compound mutants to investigate whether premature depletion of chondrocytes contributes to the reduction in bone length in mutants. The growth plate length of the mutant radius and ulna is significantly shorter than controls at newborn stages despite the absence of significant differences in bone length at this stage, and the growth plate remains shorter throughout postnatal development (Fig. 6A,B). By 6 weeks of age and beyond, differences in growth plate length are no longer statistically significant, likely due to depletion of growth plate chondrocytes in both controls and mutants by these stages (Fig. 6A,B,O-V).

All three zones of the growth plate: the reserve zone, proliferative zone, and hypertrophic zone, are established in the compound mutant, however, cellular organization of the growth plate is disorganized compared to controls. This is noted particularly in the PZ of the compound mutants. PZ chondrocytes are visualized histologically in clusters in the mutant animals, in contrast to the normal vertical columns of flattened chondrocytes seen in controls (Fig. 6D, PZ vs Fig. 6C, PZ and Fig. 6F, PZ vs Fig. 6E, PZ). While matrix secreted by the PZ chondrocytes is not uniformly organized vertically in *Hox11* compound mutants, there are no apparent differences in the amount of Safranin-O stained matrix produced compared to controls (Fig. 6D, PZ vs Fig. 6C, PZ and Fig. 6F, PZ vs Fig. 6E, PZ). Additionally, RZ chondrocytes are depleted more rapidly in mutants compared to controls; there are noticeably fewer cells in this zone by 2 weeks of age (Fig. 6H′ vs G′ and Fig. 6J′ vs I′).

**Fig. 7.**
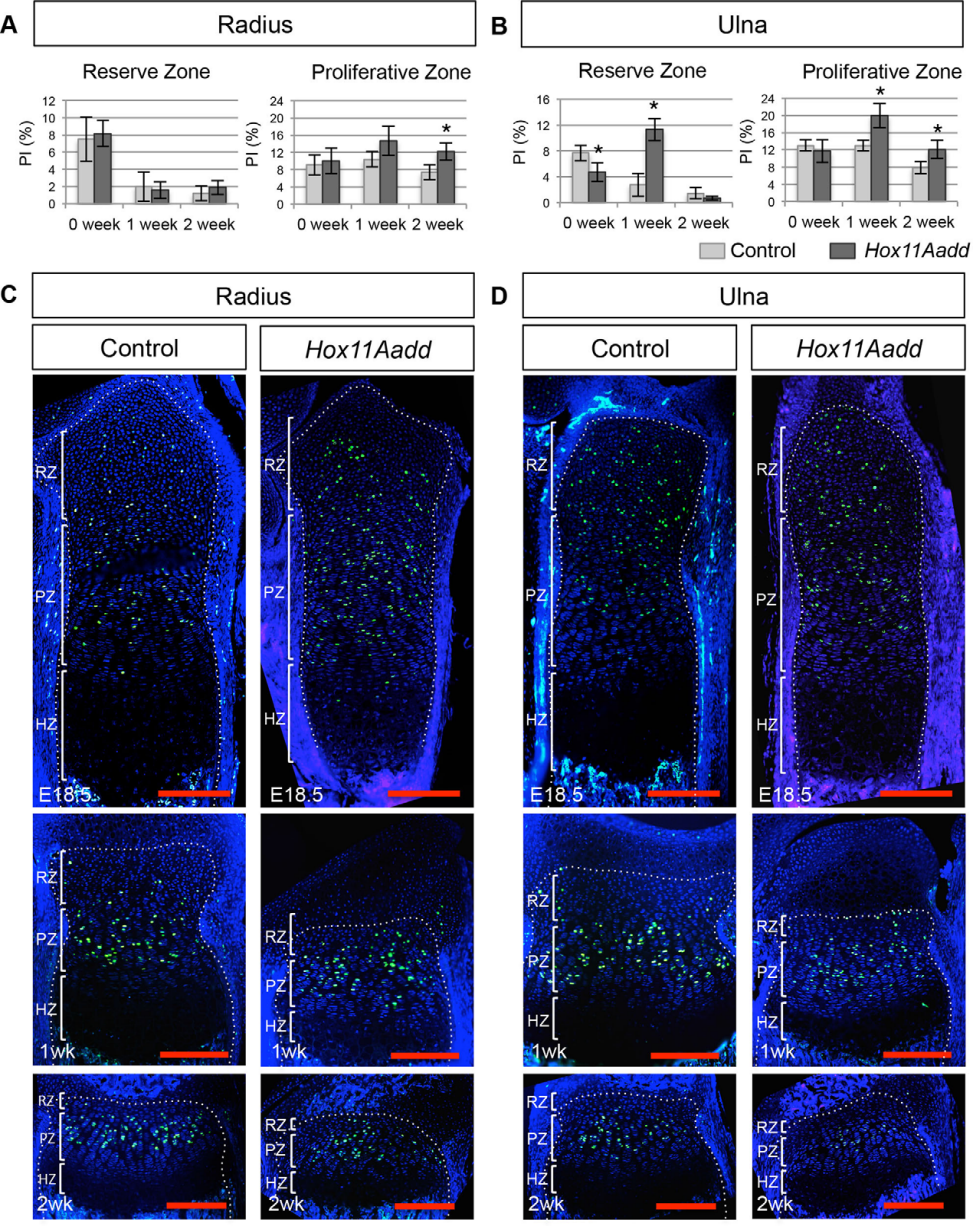
**Proliferation defects are observed in the distal growth plate of *Hox11* compound mutant forelimb zeugopod bones.** Proliferative index (PI) was calculated from total BrdU-positive nuclei relative to total nuclei in the reserve and proliferative zone at 0, 1 and 2 week in the radius (A) and ulna (B) of control (light grey) and compound mutant (dark grey) animals. Representative images of BrdU staining in the distal growth plate of the radius (C) and ulna (D) at E18.5, 1 week, and 2 weeks of control and *Hox11* compound mutant. White brackets demarcate zones of the growth plate (C-D). RZ, reserve zone; PZ, proliferative zone; HZ, hypertrophic zone. *P<0.05; error bars depict standard deviation. Scale bar 200 µm.

To examine the cause of the premature shortening of the growth plate in *Hox11* compound mutants, we examined proliferation rates in the RZ and PZ of the growth plate using BrdU labeling analysis. Since the number of RZ chondrocytes is noticeably reduced by 2 weeks of age in mutants, we focused our analysis to 0, 1, and 2 weeks of age. Proliferation in the mutant radius and ulna is higher in both the RZ and PZ than in controls, reaching statistical significance at 1 week in the ulna and 2 weeks in the radius (Fig. 7A,B). Representative images at E18.5, 1 week and 2 weeks highlight the regional differences in proliferation in the growth plates of controls and *Hox11* compound mutants (Fig. 7C,D). These data are consistent with premature depletion of growth plate chondrocytes in *Hox* mutants.

## DISCUSSION

Herein, we show that *Hox11* genes continue to be expressed and function during postnatal skeletal development. While preserving a single functional allele of *Hoxa11* or *Hoxd11* is sufficient for normal embryonic bone development, the radius and ulna of *Hox11* compound mutant animals develop abnormal skeletal morphology postnatally compared to controls. The progressive anterior bowing of the mutant radius does not appear to be due to inferior bone quality or mis-directed appositional growth. In fact, both the compound mutant radius and ulna exhibit appositional growth consistent with compensatory growth. We demonstrate that the anterior radius bowing phenotype is likely to be the result of disproportionate longitudinal growth of the radius and ulna, with the ulna lagging in growth rate compare to the radius. Uncoordinated growth leads to bowing of the faster-growing element, the radius. These data suggest that *Hox11* genes function to coordinate the longitudinal growth of the distal growth plate of the radius and ulna.

Why the radius grows faster than the ulna in the *Hox11* compound mutant is unclear. The genotype of the *Hox11* compound mutant alone cannot explain the loss of coordinated growth of the radius and ulna since the radial bowing phenotype occurs to a similar extent in both compound mutant genotypes; *Hox11Aadd* or *Hox11aaDd* (Davis et al., 1995). Embryonically, a single *Hoxa11* or *Hoxd11* allele is sufficient to support normal forelimb development. Therefore, both elements of the zeugopod clearly require *Hox11* function for proper patterning during development. These observations highlight that *Hoxa11* and *Hoxd11* genes perform similar and highly overlapping functions during embryonic development and in regulating postnatal growth. *Hoxa11* and *Hoxd11* are strongly expressed in the mesenchymal tissue surrounding the distal ends of the radius and ulna embryonically and postnatally. One hypothesis to explain why the ulna is more severely affected than the radius during postnatal growth is that there is a differential requirement for *Hox11* function in the ulna compared to the radius. Future studies utilizing conditional alleles of *Hox11* for complete loss of *Hox* function in postnatal development will further clarify the function of *Hox11* in regulating longitudinal skeletal growth.

During longitudinal skeletal growth, there is tight regulation of proliferation and chondrocyte maturation within the growth plate. The majority of proliferation occurs within the columns of PZ chondrocytes, however, RZ chondrocytes also undergo proliferation, to add new cells to the PZ (Farnum and Wilsman, 1993; Abad et al., 2002; Ballock and O'Keefe, 2003; Beier, 2005). Through postnatal development, there is a reduction in proliferation within the growth plate and a decrease in the height of the growth plate, a process termed growth plate senescence (Nilsson and Baron, 2004). Senescence is a complex process; chondrocytes within the growth plate are only capable of a finite number of cell divisions and the balance of chondrocyte proliferation and timing of maturation determines bone length (Baron et al., 1994; Gafni and Baron, 2000; Nilsson and Baron, 2004; Schrier et al., 2006). We show that there is a loss of proliferative regulation within the growth plate and premature senescence in *Hox11* compound mutants. Abnormally high levels of proliferation in both RZ and PZ chondrocytes are measured in the compound mutant ulna and radius, and longitudinal growth arrests earlier in these mutants. These observations suggest that chondrocyte maturation within the growth plate is dysregulated with loss of *Hox11* function.

We, and others, have demonstrated that *Hox11* gene expression is restricted to the perichondrium and RZ chondrocytes and that *Hox11* genes are not expressed within the PZ and HZ chondrocytes themselves (Boulet and Capecchi, 2004; Swinehart et al., 2013). Since *Hox* genes are transcription factors, this raises the possibility that *Hox* functions to maintain an undifferentiated state within the RZ chondrocyte progenitor pool and regulates when RZ chondrocytes enter the PZ and begin to differentiate. Loss of *Hox* within RZ chondrocytes may result in a loss of progenitor identity, and thus, premature differentiation and depletion of the chondrocyte progenitor pool. This hypothesis is consistent with the phenotypes observed in embryonic *Hox11* loss of function limbs. *Hoxa11^−/−^;Hoxd11^−/−^* mutant limbs are severely shortened, growth plates do not form, and chondrocyte maturation is perturbed (Boulet and Capecchi, 2004). Additionally, proliferation analysis in the *Hox11* mutant radius and ulna show proliferation throughout the cartilage anlage in contrast to control limbs where proliferation is restricted to the distal growing ends. Similar to the postnatal limb, *Hox11* genes are expressed at the distal end of the growing cartilage anlage embryonically; suggesting that *Hox11* contributes to maintaining progenitor identity and regulating the rate of proliferation and differentiation of chondrocytes in both the embryonic and adult context.

Growth plate chondrocytes undergo a well-characterized differentiation program that is common to all long bones, however, the rate of growth between growth plates of different bones vary substantially (Wilsman et al., 1996a,b). Neither systemic regulation nor local feedback loops can easily account for these differences since these factors are present in all growth plates. Our current data is consistent with a model whereby the differential growth rates across the skeleton are controlled, at least in part, by the *Hox* proteins expressed in each region. It is well documented that during development *Hox9-13* paralogs function in a highly region-specific fashion to pattern the appendages. We demonstrate that *Hox11* function remains region-specific during postnatal development and regulates long bone growth through controlling chondrocyte maturation within the growth plate.

## MATERIALS AND METHODS

### Animals

Male and female mice heterozygous for both the *Hoxa11* and *Hoxd11* null alleles and the *Hoxa11eGFP* knock-in allele were previously described (Wellik and Capecchi, 2003; Nelson et al., 2008). All procedures followed protocols reviewed and approved by the University of Michigan's Committee on Use and Care of Animals.

### qPCR analysis

Specimens were dissected in PBS on ice. Forelimbs/forelimb buds were removed and soft tissues were dissected away from the radius and ulna. The whole embryonic day (E) 12.5 limb bud or whole radius and ulna at E18.5, 1 week, 2 weeks and 4 weeks were collected into Trizol. Adult lung tissue was collected into Trizol as a control. qPCR for *Hox11* genes was performed with the following primer sets: Hoxa11F – CCTTTTCCAAGTCGCAATGT, Hoxa11R – AGGCTCCAGCCTACTGGAAT, Hoxd11F – AGTGAGGTTGAGCATCCGAG, Hoxd11R – ACACCAAGTACCAGATCCGC. Delta Ct values were calculated for each primer set relative to GAPDH at each time point assessed and normalized to expression in the adult lung which was set to ‘1’. Error bars depict standard deviation. A minimum of three animals were analyzed at each age.

### Histology, immunohistochemistry, *in situ* hybridization

All specimens were dissected in PBS on ice. Right limbs were wrapped in PBS-soaked gauze and stored at −20°C prior to µCT analysis. Left limbs were immediately processed for histology. For histological analyses, limbs were fixed for 3 days in 4% paraformaldehyde in PBS at 4°C and then decalcified in 14% EDTA for one week prior to embedding into OCT media. Newborn limbs did not require decalcification and were embedded in OCT immediately after fixation. Cryosections were collected at 16 µm through the radius/ulna.

Endogenous *Hoxa11eGFP* expression at E18.5 was visualized without the use of an antibody, however the decalcification process results in high auto-fluorescence in adult tissues and an antibody against GFP was required to visualize expression at 2 weeks. Immunohistochemical staining was performed by blocking with donkey serum and incubation with primary antibody against GFP (Invitrogen, A-11122, 1:200) overnight at 4°C. Secondary antibody was incubated at room temperature: donkey anti-rabbit Alexa Fluor488 (Invitrogen, A21206, 1:1000). Section *in situ* hybridization was performed using ^35^S-labeled riboprobes using standard techniques (Schipani et al., 1997). *Hoxd11 in situ* probes were previously described (Yallowitz et al., 2011).

For skeletal preparations, newborn animals were skinned and fixed in 95% ethanol. Fixed skeletons were stained in Alcian Blue (76% ethanol:20% acetic acid) at 37°C for 48 h, rinsed in 95% ethanol, treated with 1% KOH for 4-5 h and stained with Alizarin Red in 2% KOH for 1 h. Stained skeleton were cleared successively in 20% glycerol:1% KOH, 50% glycerol:1% KOH and 100% glycerol. Forelimbs were removed and imaged on a Leica MZ125 stereo microscope.

To visualize growth plate morphology, cryosections were stained with Safranin-O/Fast Green/Hematoxylin as previously described (Thompson et al., 2002). Images were captured on an Olympus BX-51 upright light microscope with an Olympus DP70 camera. Measurements of the total growth plate length were performed using ImageJ software (Schneider et al., 2012).

### Micro-computed tomography (µCT) analysis

Samples were scanned using an eXplore Locus SP microCT system (GE Healthcare). All specimens were scanned in water using the following parameters: voltage 80 kVp; current 80 µA; exposure time 1600 ms; voxel size in the reconstructed image 18 µm, isotropic. The data were processed and analyzed using MicroView (v 2.1.2 Advanced Bone Application; GE Healthcare Preclinical Imaging). First, the image was reoriented so that the anterior-posterior and longitudinal axes were aligned with the principal image axes. Next, the bone was manually segmented starting with a frame at the center of the bone and extending 72 µm on either side to identify a 144 µm region of interest (ROI). The tissue mineral density (TMD), cortical area, cortical thickness, and inner and outer perimeters were calculated. The total length of the radius, ulna and humerus was measured along the central curvature of the bone. A minimum of five animals were analyzed at each age.

### Bone histomorphometry

Five animals each of control and *Hox11* compound mutant were injected with xylenol orange (90 mg/kg) (Sigma), calcein (15 mg/kg) (Sigma), and alizarin complexone (30 mg/kg) (Sigma) in PBS at 2 weeks, 3 weeks and 4 weeks of age respectively. Animals were sacrificed two days following the final injection and limbs were collected. Un-decalcified bones were processed and embedded into plastic as previously published (Smith et al., 2013). 200 µm thick sections were collected, mounted to plastic slides and polished to approximately 30 µm thickness. Images were captured at 20× magnification using a Zeiss Axiovert 200M inverted microscope equipped with Apotome imaging system.

### BrdU incorporation

Timed pregnant females, 1 week, and 2 week pups were injected intraperitoneally with bromodeoxyuridine (100 mg/kg)/fluorodeoxyuridine (12 mg/kg) (Sigma) in DPBS. Pregnant females were sacrificed 2 h after injection and 1 and 2 week pups were sacrificed 4 h after injection. Specimens were dissected in PBS on ice. Forelimbs were collected and the soft tissues were removed. Limbs were fixed for 3 days in 4% paraformaldehyde in PBS at 4°C, decalcified in 14% EDTA for one week, and then washed into 70% ethanol prior to processing into paraffin. Microtome sections were collected at 7 µm through the radius/ulna. BrdU signal was visualized utilizing a BrdU immunostaining kit (Life Technologies). Total number of cells, as counted by DAPI positive nuclei, and BrdU-positive cells were counted using ImageJ software and proliferation rates were calculated as number of BrdU-positive cells divided by total number of cells. A minimum of three animals were analyzed at each age.
